# Radiation damage at the active site of human alanine:glyoxylate aminotransferase reveals that the cofactor position is finely tuned during catalysis

**DOI:** 10.1038/s41598-017-11948-w

**Published:** 2017-09-15

**Authors:** Giorgio Giardina, Alessandro Paiardini, Riccardo Montioli, Barbara Cellini, Carla Borri Voltattorni, Francesca Cutruzzolà

**Affiliations:** 1 Department of Biochemical Sciences “A. Rossi Fanelli”, Sapienza University of Rome, Rome, Italy; 2 Department of Biology and Biotechnology “Charles Darwin”, Sapienza University of Rome, Rome, Italy; 3 Department of Neurosciences Biomedicine and Movement, University of Verona, Verona, Italy; 4 Istituto Pasteur of Italy - Fondazione Cenci Bolognetti, Rome, Italy; 5 Present Address: Department of Experimental Medicine, University of Perugia, Perugia, Italy

## Abstract

The alanine:glyoxylate aminotransferase (AGT), a hepatocyte-specific pyridoxal-5′-phosphate (PLP) dependent enzyme, transaminates L-alanine and glyoxylate to glycine and pyruvate, thus detoxifying glyoxylate and preventing pathological oxalate precipitation in tissues. In the widely accepted catalytic mechanism of the aminotransferase family, the lysine binding to PLP acts as a catalyst in the stepwise 1,3-proton transfer, interconverting the external aldimine to ketimine. This step requires protonation by a conserved aspartate of the pyridine nitrogen of PLP to enhance its ability to stabilize the carbanionic intermediate. The aspartate residue is also responsible for a significant geometrical distortion of the internal aldimine, crucial for catalysis. We present the structure of human AGT in which complete X-ray photoreduction of the Schiff base has occurred. This result, together with two crystal structures of the conserved aspartate pathogenic variant (D183N) and the molecular modeling of the transaldimination step, led us to propose that an interplay of opposite forces, which we named spring mechanism, finely tunes PLP geometry during catalysis and is essential to move the external aldimine in the correct position in order for the 1,3-proton transfer to occur.

## Introduction

**Figure 1:**
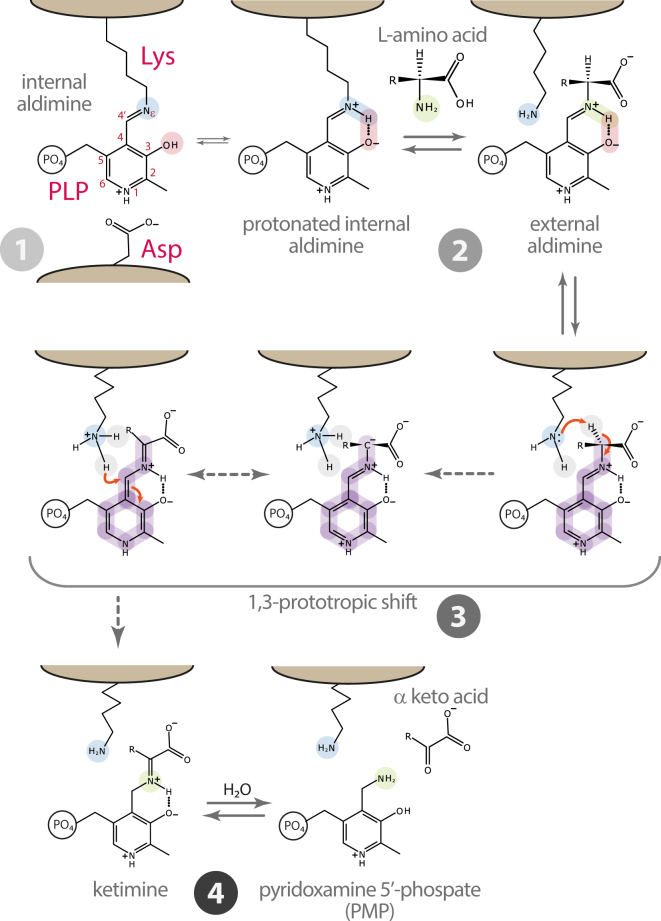
General mechanism for the first half reaction of aminotransferases. (**1**) A conserved Asp residue protonates the pyridine nitrogen of PLP (N1). (**2**) The transaldimination reaction with the amino acid substrate occurs only when the internal aldimine is protonated at Nε. (**3**) The 1,3-prototropic shift represents the rate limiting step of the half reaction. The amino group of the Lys residue acts as a base catalyst extracting the Cα hydrogen. According to the Dunathan hypothesis, co-planarity of the Schiff base bond of the external aldimine with the pyridine plane forms an extended π-system that, together with protonation of N1, allows the negative charge on Cα carbanion to be stabilized by a combination of Coulombic and resonance effects^3^. The same Lys residue serves as an acid catalyst in the protonation of C4′ completing the 1,3-proton transfer. (**4**) Hydrolysis of the resulting ketimine yields pyridoxamine phosphate (PMP) and the leaving α-keto acid.

Pyridoxal 5′-phosphate (PLP) dependent enzymes play a major role in a plethora of metabolic pathways and have long been under study^1^. In the widely accepted catalytic mechanism for the aminotransferase family, the PLP binding lysine residue also acts as a catalyst in the stepwise 1,3-proton transfer, thus converting the external aldimine to ketimine (Fig. 1)^2^. This step requires a conserved aspartate residue whose accepted role is to protonate the pyridine nitrogen of PLP (N1) to enhance the ability of the cofactor to stabilize the carbanionic intermediate^3^. However, since this aspartate residue is not invariant among PLP dependent enzymes, its role in the aminotransferase family has been questioned and is likely not limited to protonation of N1. In particular, it has been proposed that the conserved aspartate is also responsible for ground state destabilization, occurring through a geometrical distortion of the internal aldimine, and that this strain is crucial for aminotransferases catalysis^4–6^.

**Figure 2:**
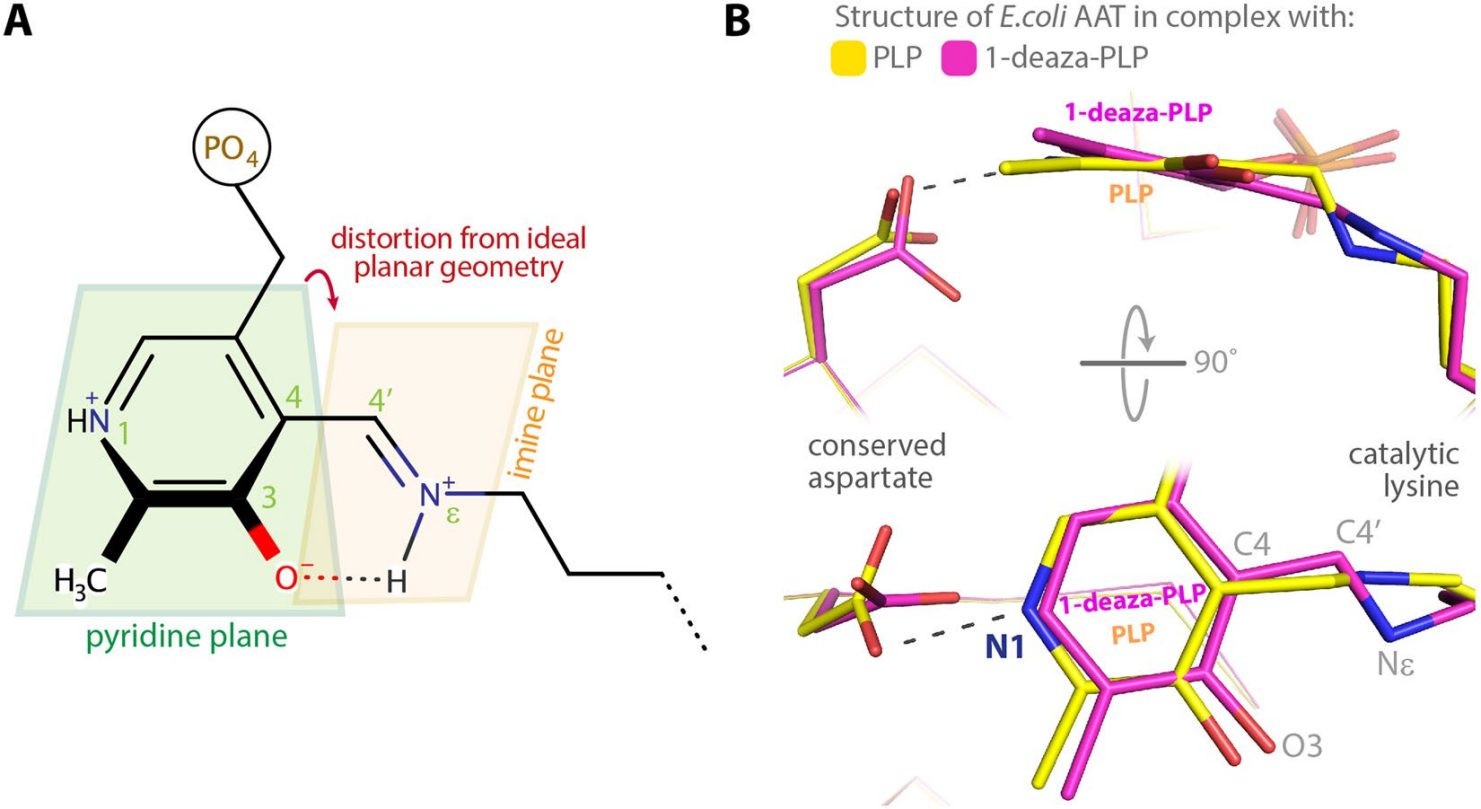
(**A**) Scheme of the distortion form ideal geometry of the internal aldimine imine plane. (**B**) Structural superposition of the internal aldimine formed by *E. coli* aspartate aminotransferase (AAT) in complex with PLP (yellow: PDB id 1 ARS) and 1-deaza-PLP (magenta: PDB id 3QN6)^7^.

The main effect of the strain is the increase of the torsion angle observed between the imine and the pyridine plane of the internal aldimine (C3-C4-C4′-Nε), which in turn destabilizes the hydrogen bond between the protonated Nε and O3 of the cofactor (Fig. 2A). Accordingly, enzymes belonging to the aminotransferase family display pK_a_ values of the Nε that are 3–4 units lower than other PLP enzymes. It has been shown that deviation from the planar geometry lowers the pK_a_ of about 2 units, while protonation of pyridine N1 accounts for the additional 2–2.5 units^4–6^.

The geometrical strain of the internal aldimine arises from the opposite torque momentum induced by the salt bridge between the conserved Asp residue with pyridine N1 of PLP and by the Schiff base linkage between Nε and C4′. This is evident by comparing the structures of *Escherichia coli* aspartate aminotransferase (AAT) in complex with PLP and with 1-deaza-PLP, the latter showing a planar conformation of the internal aldimine due to the absence of the strain^7^ (Fig. 2B). The importance of this aspartate residue in tuning the pK_a_ values of the *E. coli* AAT internal aldimine was shown in 1992^8^. Finally, Papageorgiou and co-workers showed that X-ray induced reduction of the Schiff base double bond to single bond may occur when the internal aldimine geometry is distorted^9^.

Here we present evidence of complete X-ray induced reduction of the internal aldimine double bond in the enzyme alanine:glyoxylate aminotransferase major allele (AGT-Ma; EC 2.6.1.44). By coupling this result with the crystal structure at different pH of the AGT pathogenic variant D183N, bearing the mutation of the conserved aspartate, and with a molecular dynamic simulation of the transaldimination step, we propose a novel interpretation of the conserved aspartate role.

**Figure 3:**
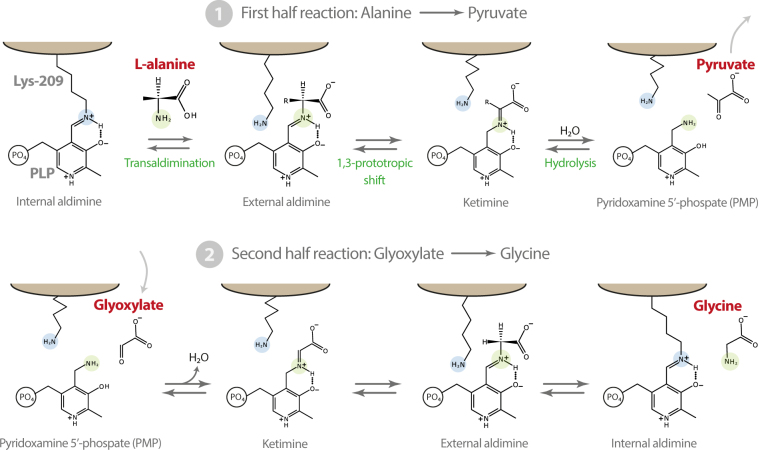
Scheme of the transamination reaction catalysed by AGT-Ma. In the first half reaction alanine is converted to pyruvate, the corresponding α-keto acid, and the amino group is transferred to the cofactor (PMP). In the second half reaction with the cosubstrate glyoxylate yields glycine and the internal aldimine between PLP and Lys-209, closing the catalytic cycle.

AGT is a hepatocyte-specific PLP dependent enzyme, belonging to the Fold Type I family, catalysing the transamination reaction between L-alanine and glyoxylate to produce glycine and pyruvate, thus leading to the removal of glyoxylate (Fig. 3). AGT normally localises in the peroxisomes and failure of its detoxification role leads to increased levels of oxalate, coming from oxidation of glyoxylate, causing calcium oxalate precipitation in the kidney and urinary tract^10,11^. Many pathogenic variants of AGT cause PH1 (primary hyperoxaluria type I), a rare autosomal recessive disorder^12–14^. Biochemical and structural data available to date indicate that several of these mutations induce very small structural rearrangement at the level of the active site that result in dramatic reduction of catalytic efficiency^10,12,14,15^.

## Results and discussion

### X-ray induced complete Schiff-base photoreduction at the active site of human AGT-Ma

Automated crystallization trials of AGT-Ma usually result in diamond shaped tetragonal crystals. However, in one droplet a single big (400 × 300 × 40 µm) yellow plate crystal was observed together with the diamond shaped ones. We collected both crystal forms at the Elettra synchrotron in Trieste. The tetragonal crystal diffracted to 2.5 Å resolution (space group P4_1_2_1_2; cell dimensions: a, b, c = 90.7, 90.7, 141.2), while the orthorhombic crystal yielded a high-resolution (1.7 Å) dataset. This crystal belonged to the P2_1_2_1_2_1_ space group (cell dimensions: a, b, c = 54.5, 101.9, 131.3 Å), with 2 molecules in the asymmetric unit (1 dimer) and with a relatively low solvent content of 40.3%, which may account for the good resolution of the diffraction data. Phases were obtained by molecular replacement using the 2.5 Å resolution structure of AGT-Ma as search model (PDB id: 1H0C ref.^16^). For this dataset, the good quality of the electron density maps allowed a straightforward model building, except for the interpretation of the density relative to the cofactor in the active site. In particular, only for this high-resolution dataset, no density for the Schiff base linkage between C4′ of PLP and the Nε of Lys-209 was observed (Fig. S1) and the best ligand to fit the density was 4′-deoxypyridoxine-5′-phosphate (PLR), which is a PLP lacking the aldehyde oxygen on C4′. Given the crystallization conditions, formation of PLR in solution or in the crystal lattice was implausible to occur. On the other hand, it is known that radiation damage might reduce the Schiff base linkage and significantly alter the internal aldimine geometry in a PLP dependent enzyme, especially if the initial geometry is distorted^9^. Therefore, a complete X-ray photoreduction of the Schiff base was hypothesized, to relax the geometrical strain of the internal aldimine. Since a complete photoreduction of the Schiff base double bond was never observed, we performed a rough calculation of the radiation dose experienced by the crystal, using the online server RadDose (http://www.raddo.se)^17^, and it resulted >5 MGy, which is a value comparable to the one reported to reduce the internal aldimine double bond of phosphoserine aminotransferase from *Bacillus alcalophilus* to a single bond (4.7 MGy)^9^.

To confirm this hypothesis, we divided the diffraction data into two datasets of 150 images each, and scaled them separately naming the first half early-dataset and the second late-dataset respectively (Table 1).

**Table 1:** Data collection and refinement statistics.

	AGT-Ma WT internal aldimine Early dataset	AGT-Ma WT reduced Schiff base Late dataset	AGT-Ma WT tetragonal crystal	AGT-Ma D183N pH 5.5	AGT-Ma D183N pH 9.0
Coordinates PDB id	5F9S	5HHY	5OG0	5LUC	5OFY
Data collection^*a*^					
Beamline	Elettra XRD1	Elettra XRD1	Elettra XRD1	ESRF ID23.2	Elettra XRD1
Space group	P2_1_2_1_2_1_	P2_1_2_1_2_1_	P4_1_2_1_2	P2_1_2_1_2_1_	P4_1_2_1_2
Cell dimensions *a, b*, *c* (Å)	54.5, 101.9, 131.3	54.5, 101.9, 131.3	90.7, 90.7, 141.2	127.6, 141.1, 256.1	89.1, 89.1, 140.7
Resolution (Å)	48.0–1.70 (1.73–1.70)	48.0–1.70 (1.73–1.70)	47.5–2.50 (2.60–2.50)	57.2–1.8 (1.83–1.80)	46.9–2.80 (2.95–2.80
R_merge_	0.08 (0.56)	0.08 (0.64)	11.6 (99.2)	0.14 (0.68)	0.09 (1.07)
CC(1/2) (%)	99.7 (75.5)	99.5 (68.3)	99.9 (93.2)	98.7 (70.7)	99.7 (95.8)
*I*/sigma *I*	10.6 (1.6)	9.0 (1.4)	19.0 (3.5)	6.2 (1.8)	20.4 (2.5)
Completeness (%)	87.0 (83.4)	95.6 (90.3)	100.0 (100.0)	94.2 (96.1)	100.0 (98.9)
Reflections					
Total observed reflections	294083 (13739)	298106 (13703)	368927 (42860)	1705990 (81187)	182942 (25020)
Multiplicity	4.2 (3.9)	3.9 (3.6)	17.5 (18.4)	4.3 (4.1)	12.5 (12.1)
B Wilson	19.8	20.2	42.5	14.4	76.5
Mosaicity	0.46	0.46	0.32	0.07	0.32
Refinement					
R_work_/R_free_	15.8/20.1	16.4/20.5	25.4 /29.4	16.3/19.2	26.2/32.4
F_o_,F_c_ correlation	0.96	0.96	0.92	0.96	0.94
Avarage *B*-factor, all atoms (Å^2^)					
Protein	22.2	22.1	55.8	13.3	92.1
Ligand	21.3 (PLP)	19.8 (PLR)	44.5 (PLP)	11.7 (PLP) 19.8 (BTB)	82.7 (PLP) 75.7 (DIO)
Water	32.0	32.1	39.7	24.5	73.0
R.M.S. Deviations					
Bond lengths (Å)	0.011	0.012	0.011	0.022	0.010
Bond angles (°)	1.150	1.156	1.463	1.598	1.395
Ramachandran: (%)					
Favoured	98.4	98.0	98.0	98.3	98.0
Allowed	1.6	2.0	1.8	1.7	1.2
Disallowed			0.2		0.8
MolProbity score^*b*^, percentile	1.48, 92^nd^	0.99, 100^th^	1.81, 98^th^	1.05, 100^th^	1.80, 100^th^

^a^Values in parentheses refer to highest-resolution shell.

^b^MolProbity score combines the clashscore, rotamer, and Ramachandran evaluations into a single score, normalized to be on the same scale as X-ray resolution; 100^th^ percentile is the best among structures of comparable resolution; 0^th^ percentile is the worst.

**Figure 4:**
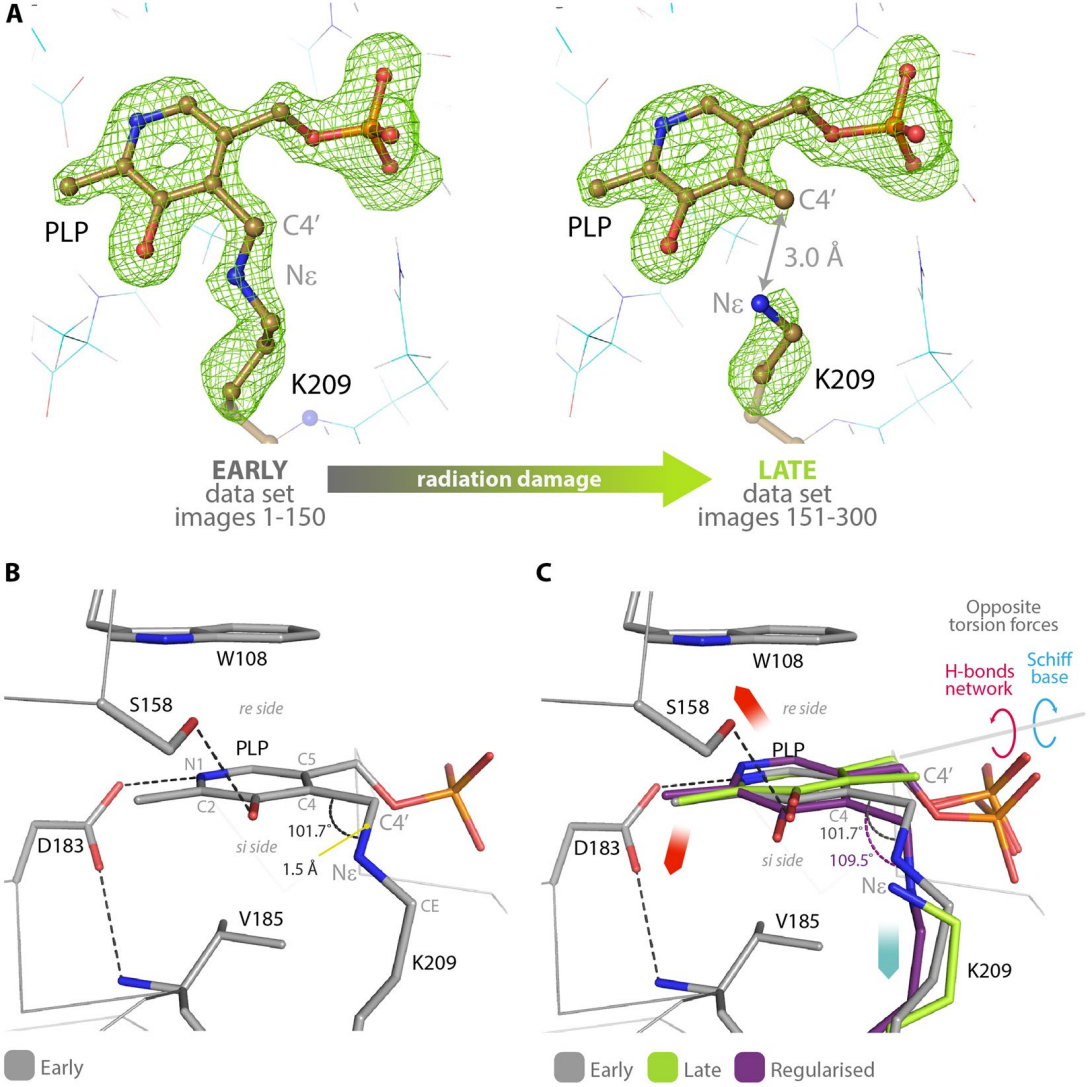
(**A**) The positive electron density of the F_o_-F_c_ omit-map (contoured at 3.5 σ = 0.38 e/Å^3^) for the early and late datasets collected on the same crystal of human AGT-Ma at 1.7 Å resolution, and the corresponding structures in ball and sticks. In the late dataset the positive electron density shows no Schiff base linkage, indicating that complete reduction of the internal aldimine was induced by radiation damage. (**B**) Stick representation of the internal aldimine in the early structure showing the key residues involved in PLP binding and geometrical orientation. (**C**) Superposition of the cofactor binding site of AGT-Ma as observed in the early structure (gray), late structure (green) and the geometrically regularized reduced internal aldimine (i.e. single bond between C4′ and Nε) (purple). The opposite torsion forces originating the geometrical strain are indicated by blue and red arrows.

Figure 4A shows the positive electron density maps for the early and late datasets, calculated by omitting both PLP and Lys-209 side chain from the refined models (see also Fig. S1). A continuous electron density between C4′ and Nε, relative to the Schiff base linkage, was observed only when the model was refined against the early dataset, while no density at all was present in the maps of the late dataset, indicating that the reduction was induced by radiation damage. The progressive reduction of the double bond electron density can be appreciated very well by refining the PLP-omit model versus 15 different datasets, each obtained by scaling 150 frames and shifting the first frame by 10 (e.g. 1–150; 10–160; 20–170; etc) (Supplementary Video S2). It must be noticed that the observed radiation damage appears to be a specific structural damage and not a global one (i.e. affecting all the residues in the crystal) since data collection statistics are almost unchanged between the two half datasets^18^.

### Comparing early and photo-reduced structures: the spring mechanism

Within the active site of AGT-Ma, the cofactor interacts with five key residues: two of them, Trp-108 and Val-185, are responsible for keeping the pyridine ring of PLP in position by a sandwich combination of π-stacking and hydrophobic interactions, while the Ser-158 H-bond with PLP-O3 and the Asp-183 salt bridge with PLP-N1, on one side, and the Schiff base linkage with Lys-209 on the other, generate two opposite torsion forces of the ring around the axis connecting C2 and C5 of PLP (Fig. 4B). Both Asp-183 and Ser-158 torque C4′ in the direction of the *re* side of the cofactor, while the Schiff base linkage pulls it in the opposite direction (*si* side) (red and blue arrows in Fig. 4C).

In the structure from early-dataset (in gray - Fig. 4B) this geometrical strain yields a significant distortion of the internal aldimine. The torsion angle between the imine and pyridine planes is 36.2°; the C4-C4′-Nε angle is 101.7° (far from 120° of an sp^2^ carbon and distorted even for an sp^3^ hybridization); finally the bond length between C4′ and Nε is 1.5 Å, equal to the average C-N distance reported for amines, while the average C = N distance of imines is 1.3 Å^19^, suggesting that in the early structure the double bond may have been reduced to a single bond.

In the late structure (in green - Fig. 4C), the Schiff bond is broken by the radiation damage, the cofactor is released from the strain and rotates 9.3° towards the *re* side, given that now the only residual torsion force is the salt bridge between the pyridine N1 and Asp-183 that acts like a spring. Consequently, when the Schiff bond is broken, the C4′-Nε distance increases from 1.5 Å, observed in the early structure, to 3.0 Å in the late one, the optimal distance for the 1,3-protortropic shift to occur^20^. On the contrary, a geometrical regularization of the reduced form of internal aldimine (in purple) tilts the cofactor towards the *si* side, indicating that even considering the C4′-Nε as a single bond the torsion induced by the Schiff base pulls the cofactor in the opposite direction with respect to the salt bridge between Asp-183 and pyridine N1.

Taken together these results suggest that the role of the conserved Asp residue in the active site of AGT-Ma has been underestimated. Not only it serves to protonate the pyridine N1 and is responsible for ground state destabilization induced by the geometrical strain, but also forces the cofactor to assume the correct position during both the transaldimination step and the 1,3-prototropic shift, acting like a spring. In particular the photo-reduced structure (late) shows that, once the Schiff base is reduced, the interaction with Asp-183 alone tilts the cofactor and brings it in a productive distance (about 3 Å) for the subsequent 1,3-prototropic shift to take place^20^. In other words, the tilt of the cofactor pyridine plane observed upon external aldimine formation is very likely induced by the spring-interaction with the conserved aspartate. Therefore, with this novel perspective, we solved and analysed the structure of the D183N pathogenic variant of AGT-Ma and reconsidered some of our previously published data on other variants of AGT-Ma, including D183N and S187F^12,15^.

### Replacement of the conserved Asp-183 with Asn reliefs the strain slowing down the enzyme

D183N is a pathogenic variant of AGT that causes Primary Hyperoxaluria Type I (PH1), a severe disorder of glyoxylate metabolism. Although the mutation is conservative, the variant shows a 2.3 × 10^4^ fold reduction of the catalytic efficiency, whereas the affinity for PLP is almost unchanged^12^. Similar results were obtained in a mutational study of D222 of *E. coli* AAT^8^. Indeed the asparagine residue is unable to protonate PLP at N1, but may this account for such a dramatic effect? Following our novel hypothesis, and to understand if the low catalytic efficiency may be due to a non-native geometrical strain, we have solved the structure of the D183N mutant at 1.8 Å resolution and at a similar pH value (pH 5.5 vs 5.0) of the wild type.

**Figure 5:**
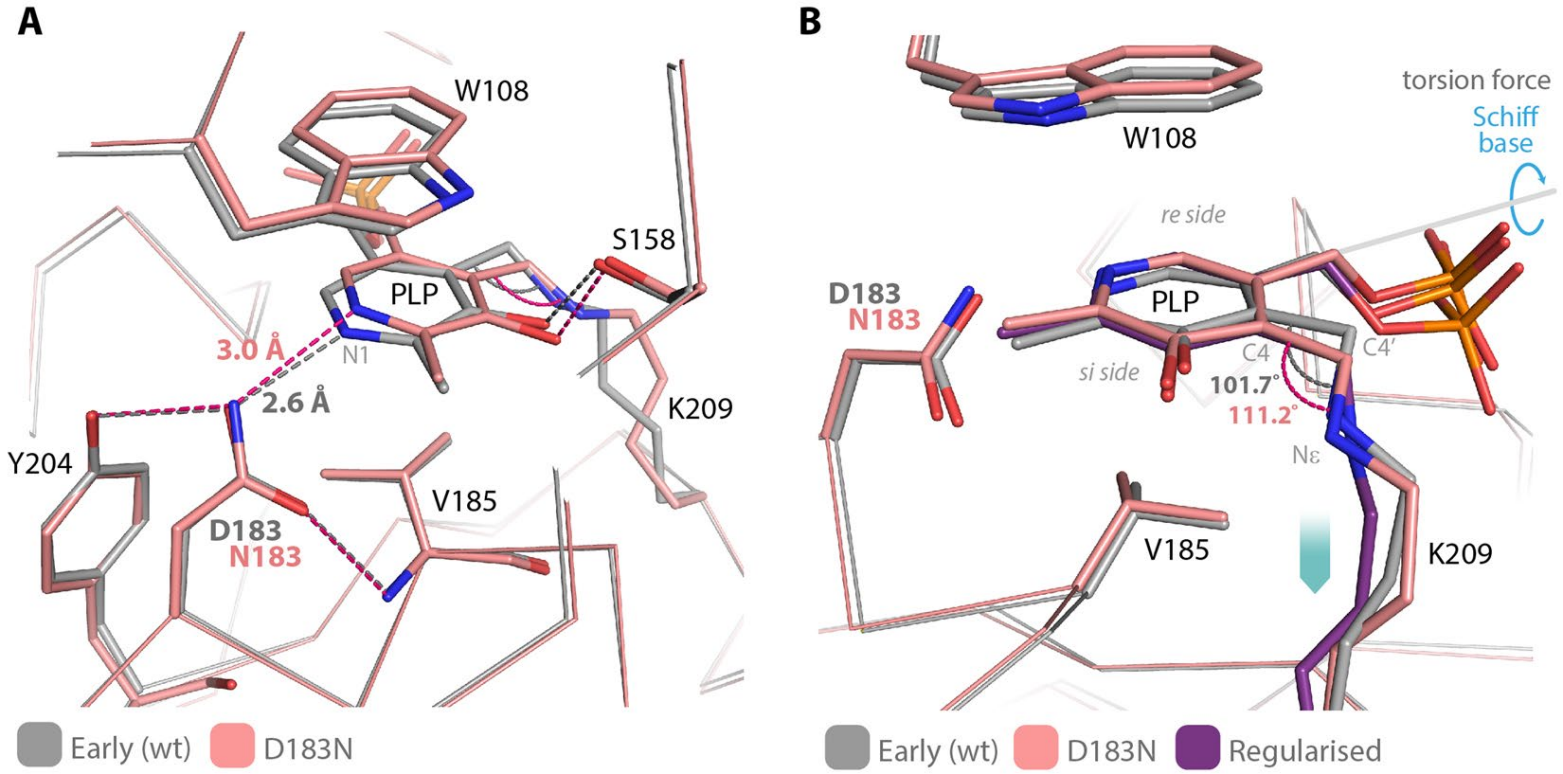
(**A**) Superposition of the cofactor binding site of wild type AGT-Ma (early structure - gray) and D183N mutant (pink). All the native interactions with PLP are conserved, except for the salt bridge between pyridine N1 and Asp-183, which is replaced by an H-bond between the amino group of the asparagine side chain and the lone pair of N1. (**B**) The internal aldimine of the D183N mutant (pink) superposes with the geometrically regularized reduced internal aldimine (i.e. single bond between C4′ and Nε) (purple), indicating that the modified H-bond network is unable to balance the Schiff base pulling force. Blue arrows indicate the residual torsion force induced by the Schiff base linkage.

The structure of D183N shows that the interaction of the asparagine side chain with N1 is loosen, going from a 2.6 Å distance in the wild type to 3.0 Å (Fig. 5). As a consequence of the strain relaxation, the torsion angle between the imine and pyridine planes is reduced by 10° (from 36.2° to 26.3°) and the cofactor tilts in the direction of the Lys (*si* face), assuming a conformation that superposes very well with the geometrically optimized reduced internal aldimine. While the wild type enzyme at pH > 7.5 shows spectral changes, which can be interpreted as deprotonation of the internal aldimine, in the mutant the internal aldimine is protonated over a wide range of pH values (5.0–9.2) (Fig. S3). This effect is also a consequence of the strain relaxation, which increases the stability of the protonated species. Accordingly when we solved the structure of D183N at pH 9.0, although at a lower resolution (2.8 Å - PDB id: 5OFY), the cofactor showed the same tilt that we observed in the structure at pH 5.5. Within the experimental error, the H-bond distance between the mutated side chain and PLP-N1 is also the same (pH 5.5 = 3.0 Å; pH 9.0 = 3.2 Å) (Fig. S3).

The structure of D183N indicates that the geometry of PLP must be finely tuned in order for catalysis to occur. Clearly, the H-bond between the mutated Asn amino group and the deprotonated pyridine N1 is not as strong as the salt bridge present in the wild type enzyme. Therefore, in the mutant the opposite torsion effect is uncoupled and the spring mechanism, responsible for the tilt of the PLP pyridine plane during the transaldimination step, is lost. The strain release of the D183N mutant is also likely responsible for the slight increase in thermal stability observed for this variant^12^, and for its apparent resistance towards radiation damage, given that the internal aldimine of the mutant seems not to undergo x-ray induced-reduction both at acidic and alkaline pH (Fig. S4).

### The spring mechanism is uncoupled also in the S187F pathogenic variant

**Figure 6:**
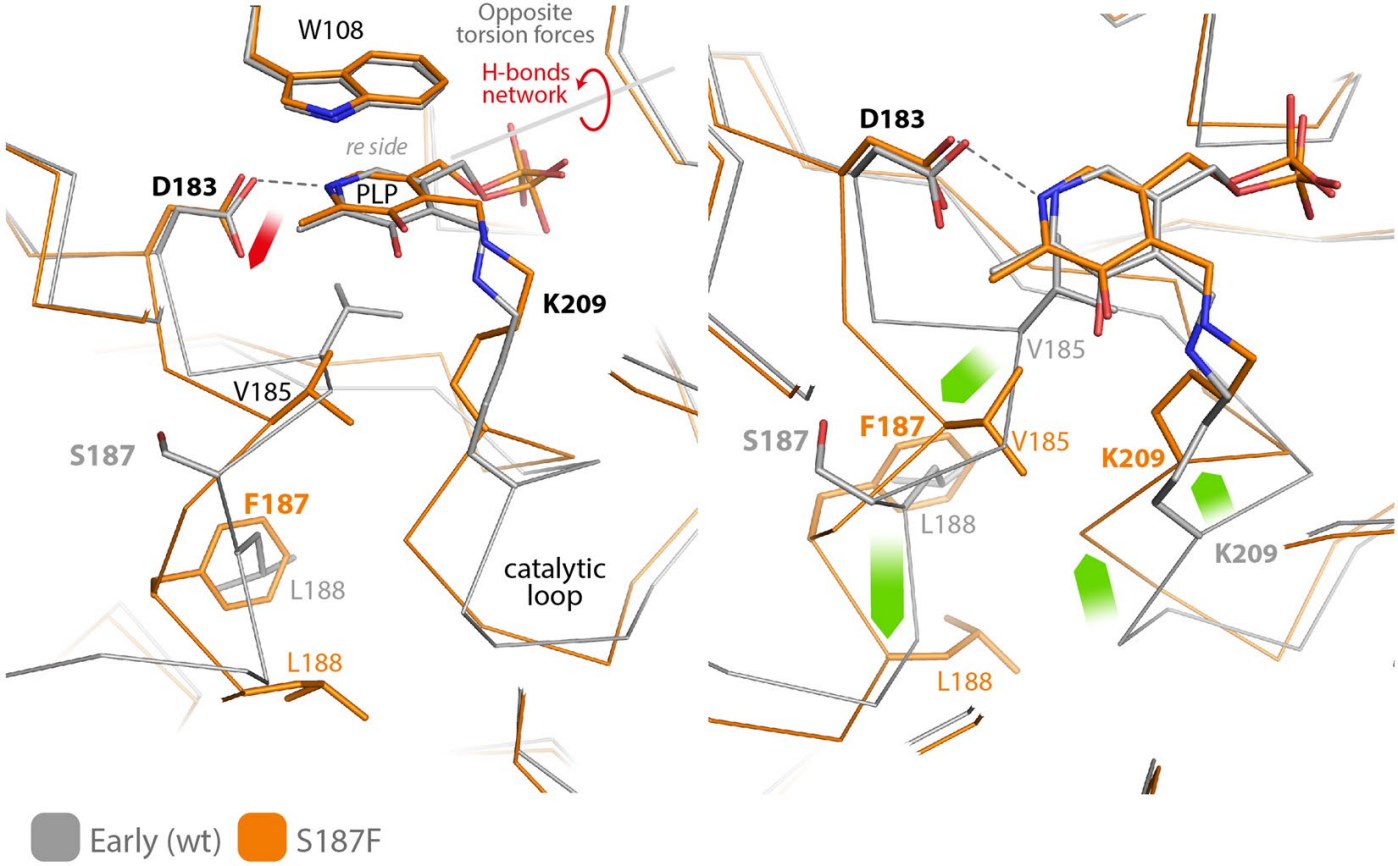
Superposition of the internal aldimine structures of wild type AGT-Ma (early structure - gray) and S187F mutant (orange; PDB id 4I8A ref.^15^). Green arrows map the backbone rearrangements induced by the mutation. The main effect of the rearrangement is the new position of Lys-209 Cα that is 1.2 Å closer to the C4′ of PLP. This movement disrupts the pulling force induced by the Schiff base linkage in the wild type, uncoupling the spring mechanism. Accordingly, the residual pulling force due to the salt bridge between Asp-183 and pyridine N1 (red arrow) tilts the cofactor plane towards the *re* side.

The biochemical and catalytic properties of S187F can also be analysed in light of an uncoupling of our novel spring mechanism. Compared to wild type AGT-Ma, this variant shows an increased binding affinity for PLP and a lower K_m_ for L-alanine, but exhibits a 1.3 × 10^2^ fold reduction of catalytic efficiency^12^. The structure of this mutant was recently solved^15^; with respect to the wild type structure, the mutated side chain of Phe-187 swaps its position with Leu-188, in order to interact with a buried hydrophobic cluster, driving back the loop harbouring Val-185, and leaving enough space for the backbone of the loop containing Lys-209 to move towards the cofactor (Fig. 6). This rearrangement reduces the distance between PLP-C4′ and the Cα of Lys-209 from 6.3 to 5.1 Å, tilting the pyridine plane towards the *re* side.

Also in the S187F variant, as previously shown for the D183N mutant, the observed decrease in the catalytic efficiency can be ascribed to an unbalance of the spring mechanism caused, in this case, by the movement of Lys-209, which no longer pulls the cofactor towards the *si* face. Accordingly in S187F the C4-C4′-Nε angle is 108.2° and the dihedral angle between the pyridine and imine planes is reduced to 29°.

## Conclusions

**Figure 7:**
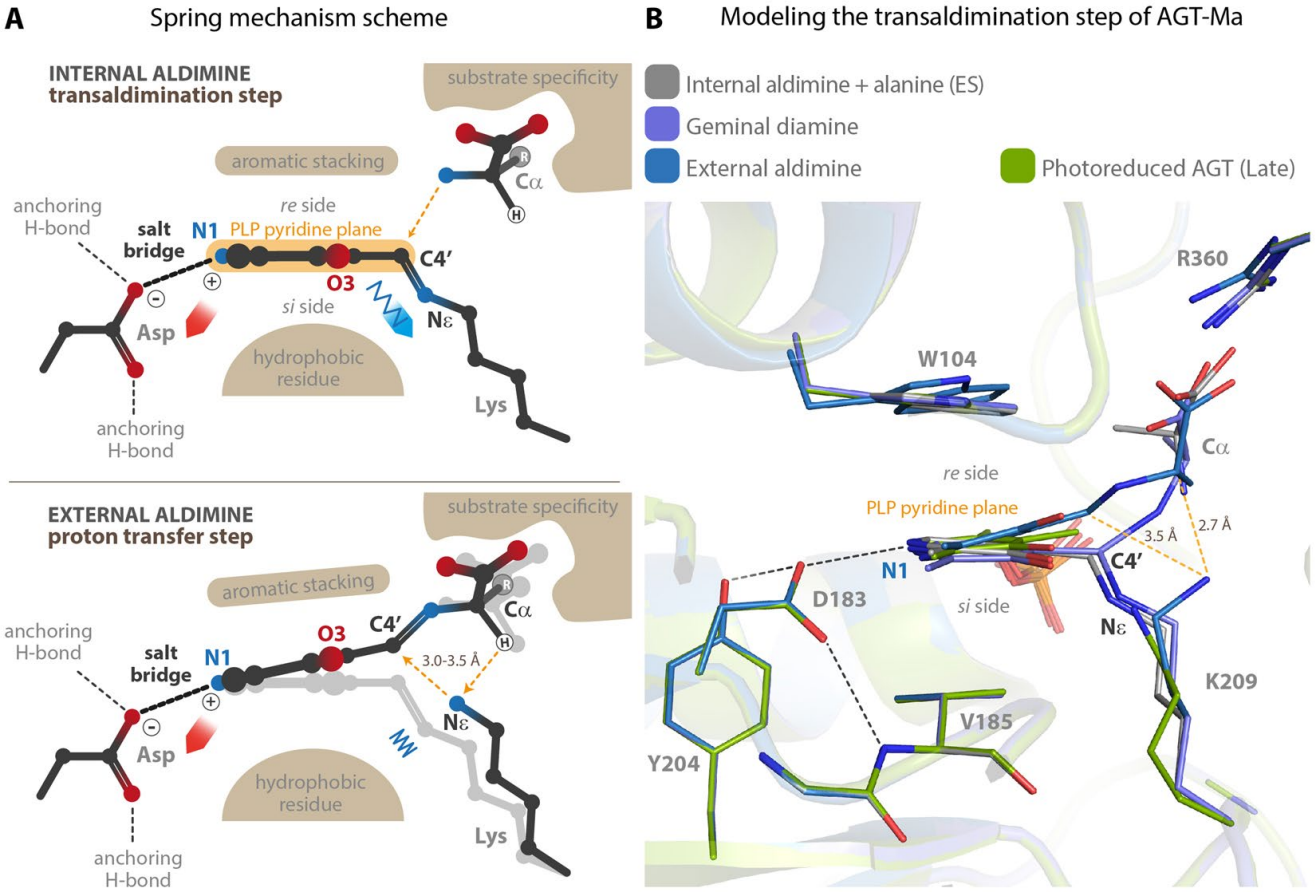
(**A**) Generic scheme of the spring mechanism. The pyridine plane of PLP is kept in position by an aromatic stacking interaction on the re side and a hydrophobic interaction on the si side. The conserved aspartate forms a salt bridge with PLP-N1, while the Schiff base with the catalytic lysine pulls the cofactor in the opposite direction. These opposite forces originate the geometrical strain of the internal aldimine. When the external aldimine is formed, the spring is released and the cofactor tilts towards the re side. This movement positions the PLP-C4′ and the amino acid substrate Cα at the correct distance, between 3.0 and 3.5 Å, from the amino group of the catalytic lysine in order for the proton transfer to take place. (**B**) Structural superposition of the models of the species involved in the transaldimination step of AGT-Ma, shown in the same orientation of the scheme in panel A. In particular: gray - internal aldimine (early dataset structure) + alanine (Michaelis complex ES); light purple - Geminal diamine; blue - external aldimine. The entire step is mapped in Supplementary Video S5. The late dataset structure (green) is also shown to highlight that the tilt of the pyridine plane that follows photoreduction of the Schiff base is comparable with the one observed in the modeled external aldimine.

Among the aminotransferase family of PLP dependent enzymes the role of the conserved aspartate residue has been long under study. Following the Dunathan hypothesis, it was initially considered necessary to protonate pyridine N1, thus enhancing the cofactor ability to stabilize the quinonoid intermediate formed after the heterolytic cleavage of one of the Cα bonds of the external aldimine^3^. However this step, which is common to all PLP-catalyzed reactions, is not rate limiting in the aminotransferase reaction^21^. Then, the work from the group of Kagamiyama showed that the internal aldimine of *E. coli* AAT suffers of a significant geometrical distortion. This strain, induced by the conserved aspartate, destabilizes the protonated internal aldimine and reduces the energy gap between E + S and the Michaelis complex ES, thus increasing the k_cat_/K_m_ value^4–6,8,22^. Our serendipitous results add another piece to this puzzling story, which may further clarify the role of the conserved Asp in aminotransferases. In the Michaelis complex of the first half reaction the C4′ atom of PLP must be at a productive distance from the amino group of the substrate in order for transaldimination to take place; however, once the external aldimine is formed, C4′ has to move away from Lys-209 Nε, which must catalyze the following proton transfer from Cα to C4′. This is the rate limiting step in the aminotransferase catalytic mechanism^21^. Our results shows that two forces generated by the salt bridge between the conserved aspartate residue and the pyridine N1 atom, and by the Schiff base linkage act as opposite springs and are responsible for the fine tilting adjustments of the pyridine plane of PLP (Fig. 7A). Our hypothesis is that in AGT-Ma these small back and forward movements induced by the strain allow the C4′ atom to be positioned at the productive distance of about 3 Å in order for both the transaldimination and the 1,3-prototropic shift to take place during catalysis. Thus, we performed a molecular modeling of the transaldimination reaction intermediates and the result is in good agreement with our hypothesis, with Lys-209 Nε ending at 3.5 and 2.7 Å from C4′ and Cα respectively (Fig. 7B). An animation through 90 states of the transaldimination step shows that the PLP pyridine plane tilts immediately after the Nε-C4′ Schiff bond breaking (Supplementary Video S5).

Each of the analyzed mutants of AGT unbalances the spring mechanism by disrupting one of the opposite forces, and both display a remarkable reduction in catalytic efficiency. In the D183N variant the H-bond replacing the salt bridge isn’t strong enough to keep the internal aldimine under strain and the pyridine plane is tilted towards the *si* side, whereas in the S187F variant the salt bridge is conserved but the counter pulling force of the Schiff base linkage is lost, due to the rearrangement of the Lys-209 loop, and the pyridine plane of the internal aldimine is tilted towards the *re* side. Intriguingly, the opposite geometrical defects of the internal aldimine in the two mutants appear to be reflected in their ability to bind the substrate and to form the external aldimine, with D183N displaying a K_m_ for alanine of 140 mM, which is five time higher with respect to wild type AGT-Ma (31 mM), while S187F shows a K_m_ of 12 mM that is even lower than the wild type^12^. Indeed, in the Michaelis complex of D183N the distance between the PLP-C4′ and the alanine amino group is likely increased, due to the tilted pyridine plane, whereas the same distance is reduced in S187F. Consequently, with respect to the wild type, the transaldimination step should be unfavored in D183N and favored in S187F. On the contrary, the 1,3-prototropic shift is likely unfavored in both variant, due to the uncoupling of the spring mechanism. Moreover, since the D183N variant is also unable to protonate the PLP-N1, and the loss in catalytic efficiency of this mutant is double with respect to S187F, this suggests that the contribution of PLP protonation at N1 and of the spring mechanism are almost equivalent and that the conserved aspartate serves to achieve both. It would be expected that, in order to serve in the spring mechanism, the position of the aspartate side chain should be firmly anchored by additional H-bonds (Fig. 7). Interestingly, mutation of the residues involved in H-bonding the aspartate was shown to affect the rate of catalysis of AAT from *Sus scrofa* ^23^. The authors correlate the effects of mutations to the ability of the aspartate to protonate PLP-N1. However, these second shell residues are very variable among the aminotransferase superfamily, and therefore we think that these results may also be interpreted in light of the spring mechanism (Fig. S6). Finally, it is important to notice that the distance between the catalytic lysine Cα and PLP-C4′ is on average 1 Å higher in aminotransferases than in other PLP dependent enzymes. Therefore, both components of the spring mechanism, the aspartate residue and the distorted Schiff base linkage, are peculiar of the aminotransferase family^4,9^. These observations suggest that, in order to use the lysine residue also as base catalyst in proton transfer, this family of PLP dependent enzymes may have evolved a unique spring mechanism to adjust the cofactor position during the first half reaction. Therefore, the lower stability and pK_a_ of the internal aldimine as well as the higher K_d_ for PLP of aminotransferases with respect to other PLP dependent enzymes, which are effects of the geometrical strain, may represent the evolutionary cost that these enzymes have to pay in order to achieve reaction specificity.

## Materials and methods

### Protein expression and purification

AGT-Ma wild type and D183N mutant were expressed and purified as described in refs^11,12^ respectively.

### Crystallization and data collection

#### AGT-Ma wild type

Both tetragonal crystals (150 × 200 × 200 µm) and the single yellow orthorhombic crystal (approximately 300 × 200 × 40 µm) were obtained by hanging drop vapour diffusion method, at 21 °C by mixing equal volumes (1 µl) of a protein solution, containing AGT‐Ma holo (8 mg/ml); NH_2_OH 3.5 mM; Jeffamine^®^ (Hampton) 3.5% v/v; 18 mM potassium phosphate pH 7.4; 20 mM Hepes pH 7.4; glycerol 5% v/v, and reservoir solution, containing polyethylene glycol (PEG) 6 K 12% v/v, 0.1 M MES pH 5.0. The crystals were cryo-protected by soaking in the mother liquor solution containing 25% 2-methyl-2,4-pentanediol (MPD) and flash freezed. Data were collected at the ELETTRA synchrotron of Trieste (XRD1 beamline) at a wavelength of 1.0 Å. Data were indexed and integrated with XDS^24^. For the tetragonal crystal: 250 frames (oscillation 1.0°) yielded a 2.5 Å resolution complete dataset. The space group was P4_1_2_1_2 with the following unit cell parameters; a = b = 90.7, c = 141.2, a solvent content of 64% and 1 monomer in the asymmetric unit. For the orthorhombic crystal a total of 225 degrees (300 frames with oscillation of 0.75°) were collected at 1.7 Å resolution. The space group was P2_1_2_1_2_1_ with the following unit cell parameters; a = 54.5, b = 101.9, c = 131.3. The final models consisted of 2 molecules (1 dimer) per asymmetric unit. To uncover the photoreduction event, the first 300 frames of this dataset were divided into two complete dataset at 1.7 Å resolution (1 to 150 early dataset; 151 to 300 late dataset) and scaled separately. All data were scaled with the software tools from the CCP4 suite ref.^25^. Data were phased by molecular replacement in Molrep^26^ using the 2.5 Å resolution AGT structure (PDB id; 1H0C ref.^16^) as search model. Cycles of model building and refinement were carried out with Coot^27^, Refmac^28^ and Phenix-Refine^29,30^; model geometry was validated with MolProbity^31^. The statistics for data collection, model building and refinement are reported in Table 1. Coordinates have been deposited to the Protein Structure Database with accession number: 5F9S, 5HHY and 5OG0 respectively for the early and late dataset of the orthorhombic crystal and for the tetragonal crystal.

#### AGT-Ma D183N at pH 5.5 and pH 9.0

Diamond shaped yellow crystal of the mutant (approximately 300 × 300 µm) were obtained in one week by sitting drop vapour diffusion method, at 21 °C by mixing equal quantities (0.4 µl) of a protein solution - containing AGT‐Ma-D183N holo (15 mg/ml); 40 mM potassium phosphate pH 7.4, 60 mM Hepes pH 7.0 and a 1.6 fold excess of PLP - with reservoir solution containing: i) polyethylene glycol (PEG) 10 K 17% w/v, 0.1 M Bis-Tris buffer pH 5.5 and 0.1 M ammonium acetate; ii) dioxane v/v 2%, 0.1 M bicine pH 9.0, PEG 20 K w/v 10%. The crystal were cryo-protected by soaking in the mother liquor solution containing 25% glycerol before flash freezing. For the crystal grown at pH 5.5, a total of 107 degrees (oscillation 0.1°) were collected at the ESRF synchrotron of Grenoble (ID23.2 beamline) at a wavelength of 0.87 Å, yielding a 1.8 Å resolution dataset. Data were indexed and integrated with XDS^24^. The space group was P2_1_2_1_2_1_ with the following unit cell parameters; a = 127.6, b = 141.1, c = 256.1 and eight molecules (4 dimers) per asymmetric unit. For the crystal grown at pH 9.0, 180 frames (oscillation 1.0°) were collected at the ELETTRA synchrotron of Trieste (XRD1 beamline) at a wavelength of 1.0 Å, yielding a 2.8 Å resolution dataset. Data were indexed and integrated with XDS^24^. The space group was P4_1_2_1_2 with the following unit cell parameters; a = b = 89.0, c = 140.8, and one molecule in the asymmetric unit. Data were scaled with the software tools from the CCP4 suite^25^, and phased by molecular replacement in Molrep^26^ using the 1.7 Å resolution wild type AGT-Ma dimer (PDB id; 5F9S this work) as search model. Cycles of model building and refinement were carried out with Coot^27^, Refmac^28^ and Phenix-Refine^29,30^; model geometry was validated with MolProbity^31^. The statistics for data collection, model building and refinement are reported in Table 1. Coordinates have been deposited to the Protein Structure Database with accession number: 5LUC and 5OFY for the crystals at pH 5.5 and pH 9.0, respectively.

### Molecular modeling studies

The coordinates of the early structure of human AGT-Ma (PDB code: 5F9S; this work) was used as a starting point to generate the transaldimination step species bound to the enzyme by means of energy minimization, using the BIOPOLYMER package from InsightII (V.2000, MSI, Los Angeles, USA). PLP was removed from 5F9S and the other moieties were then positioned into the active site of 5F9S, initially following the binding mode of PLP. Water molecules of the active site were included into the system. Atomic potentials, partial and formal charges were defined using the Cff91 forcefield, and it was verified that the proper values had been assigned. The conformational space of Lys209 was initially explored using the rotameric library of the HOMOLOGY package from InsightII, and the energetically most favorable rotamer was chosen and applied to the side-chain for further energy minimization. The minimization protocol adopted is already described^11^. Briefly, all atoms except added hydrogens were fixed to allow hydrogens to adjust to the atomic environment, with 1000 steepest descents steps, until the maximum energy derivative was less than 41.8 kJ mol^−1^Å^−1^. Then main-chain atoms were fixed and side-chains of every residue comprised in a sphere of 8 Å from the pyridine ring were subjected to a gradually decreasing tethering force (from 4180 kJ Å^−1^ to 418 kJ Å^−1^) using conjugated gradients, until maximum derivative was less than 4.18 kJ mol^−1^Å^−1^. Finally, a decreasing tethering force was applied using conjugated gradients, until the maximum derivative was less than 0.4 J mol^−1^Å^−1^. The same minimization protocol was used to energy minimize the conformations obtained during the proposed reaction mechanism, by using the MoMA-LigPath server (http://moma.laas.fr)^32^. Discover 2.9 and Analysis package of InsightII were used for minimization.

## Electronic supplementary material

Supplementary Information

Supplementary Video S2

Supplementary Video S5
