# Supplementary material for: Radiation damage at the active site of human alanine:glyoxylate aminotransferase reveals that the cofactor position is finely tuned during catalysis
Source: Sci Rep. 2017 Sep 15;7:11704. doi: 10.1038/s41598-017-11948-w (PMC5601474; doi:10.1038/s41598-017-11948-w)
Supplement: Supplementary file 1 — Supplementary Information [file 41598_2017_11948_MOESM1_ESM.pdf]

Supplementary information

Radiation damage at the active site of human alanine:glyoxylate aminotransferase reveals that the cofactor position is finely tuned during catalysis

Giardina G, Paiardini A, Montioli R, Cellini B, Borri Voltattorni C, Cutruzzolà F.

Supplementary Figure S1.

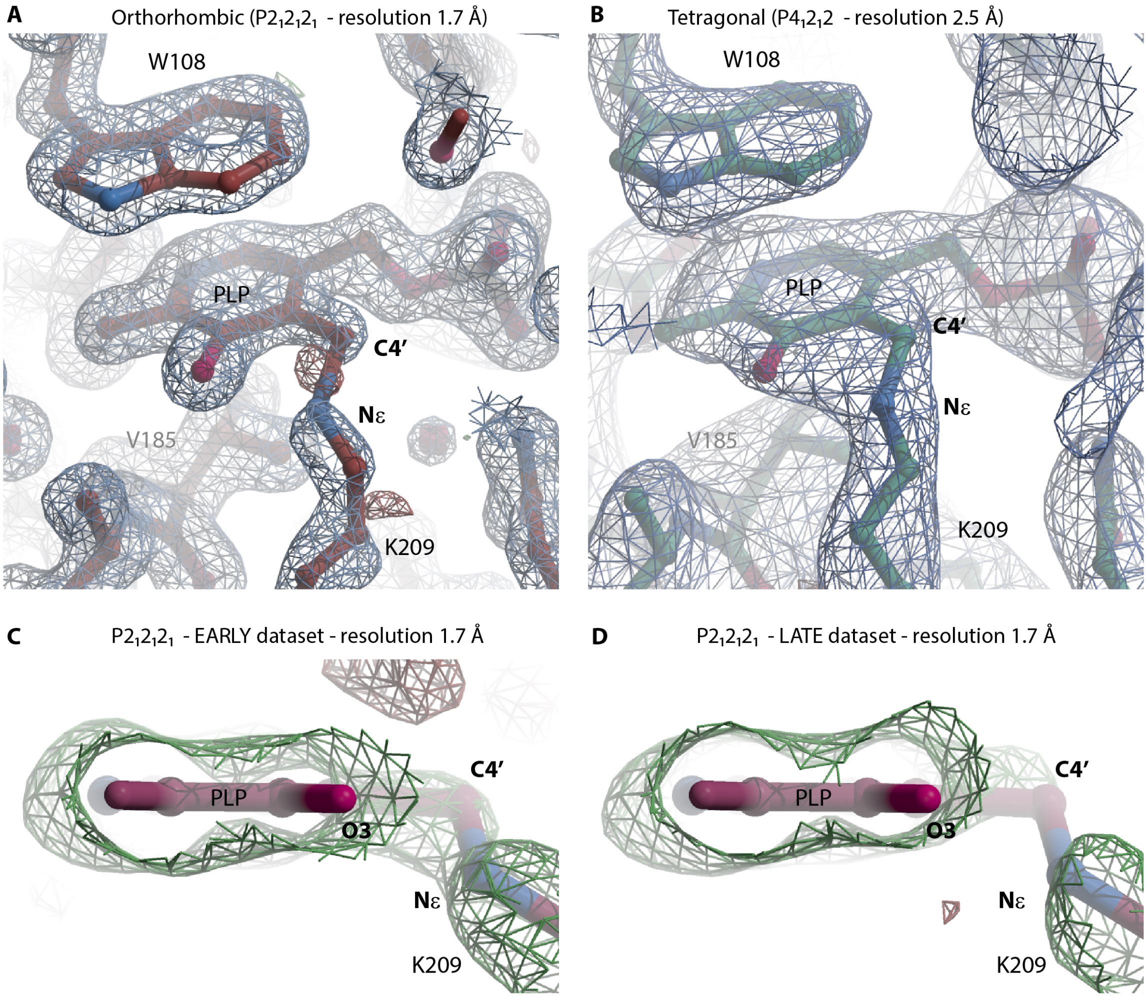

Figure S1. Electron density maps (in blue the $2F_o$-F_c and in red F_o-F_c contoured at 1.5 and 3.2 σ, respectively) calculated by refining the AGT-Ma wt model against; (**A**) the orthorhombic initial dataset at 1.7 Å resolution (i.e. frames 1 to 300); (**B**) the tetragonal 2.5 Å resolution dataset. Lack of electron density was observed in the region corresponding to the Schiff base bond between PLP-C4' and the amino group of Lys-209 only for the orthorhombic crystal. C) and D) $2F_o$-F_c maps contoured at 3.5 σ calculated by refining the AGT-Ma wt model lacking PLP against the first half dataset (Early) and the second half dataset (Late). The maps are clipped at the level of the PLP pyridine plane and shown in the same orientation. The In the Late dataset the positive electron density of the plane is clearly tilted and no density is observed for the Schiff base bond. The Early structure (5F9S) is shown in both panels.

Supplementary Video S2.

The movie shows the progressive reduction of the electron density corresponding to the internal aldimine Schiff bond. It is an animation through fifteen different F_o-F_c electron density maps, contoured at 3.5 σ. Each map was calculated by refining the AGT-Ma structure (omitting both the PLP and Lys-209 side chain) against fifteen different datasets obtained by scaling 150 diffraction image and shifting the starting frame by 10 batches per dataset (e.g. 0-150; 10-151; . . . ;150-300).

Supplementary Figure S3.

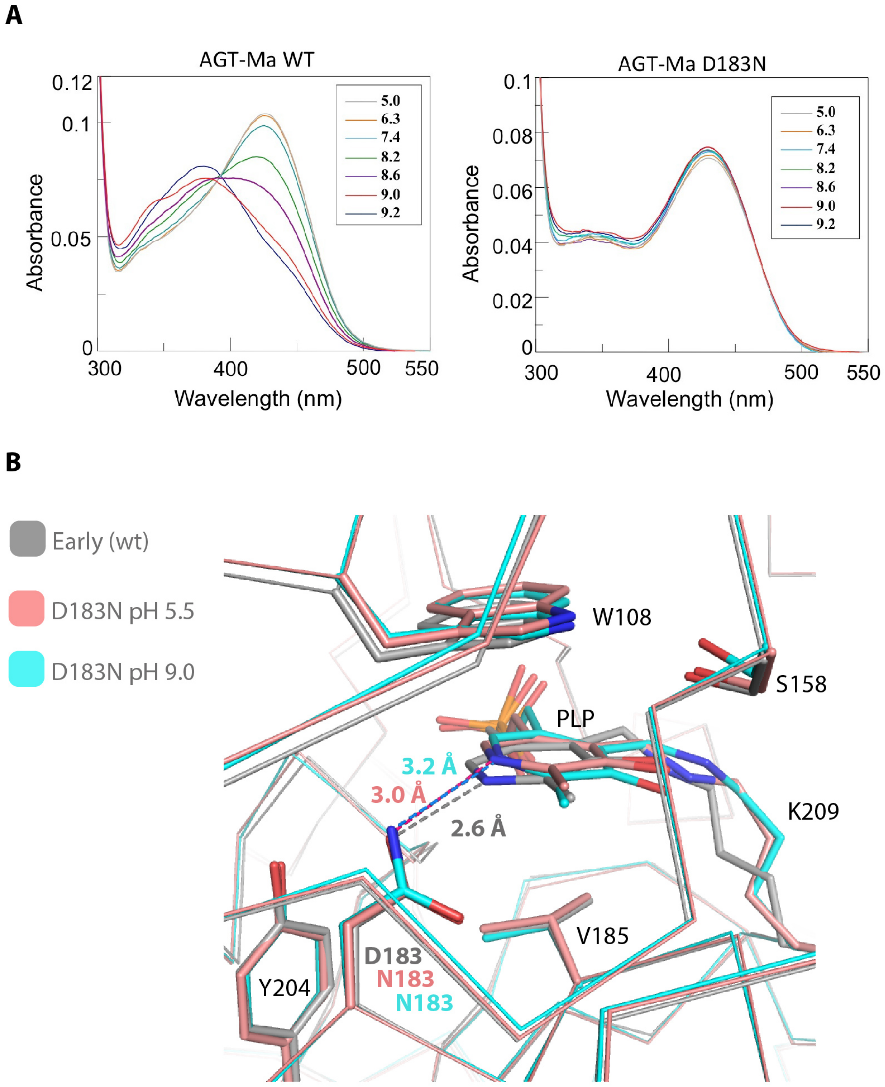

Figure S3. A) UV-vis spectra of AGT wild type compared to AGT-D183N at different pH. The protonated internal aldimine peaks at 425 nm for the wild type and at 430 nm for D183N. For the wild type enzyme the internal aldimine is in the protonated state at pH < 7.5, while it is mainly deprotonated at alkaline pH values (peak at 380 nm). On the contrary, in the same pH range the internal aldimine of the mutant remains in the protonated state. Absorption spectra were registered with a Jasco V-550 spectrophotometer upon incubation of 10 µM enzyme at 25°C for 15 min in 50 mM Bis-Tris-propane over the pH range 5.0-9.2. **B)** Superposition of the structures of the mutant D183N solved at different pH (5.5 *pink*, and 9.0 *cyan*) showing that the tilt of the PLP pyridine plane with respect to the wild type structure (*gray*) is retained.

Supplementary Figure S4.

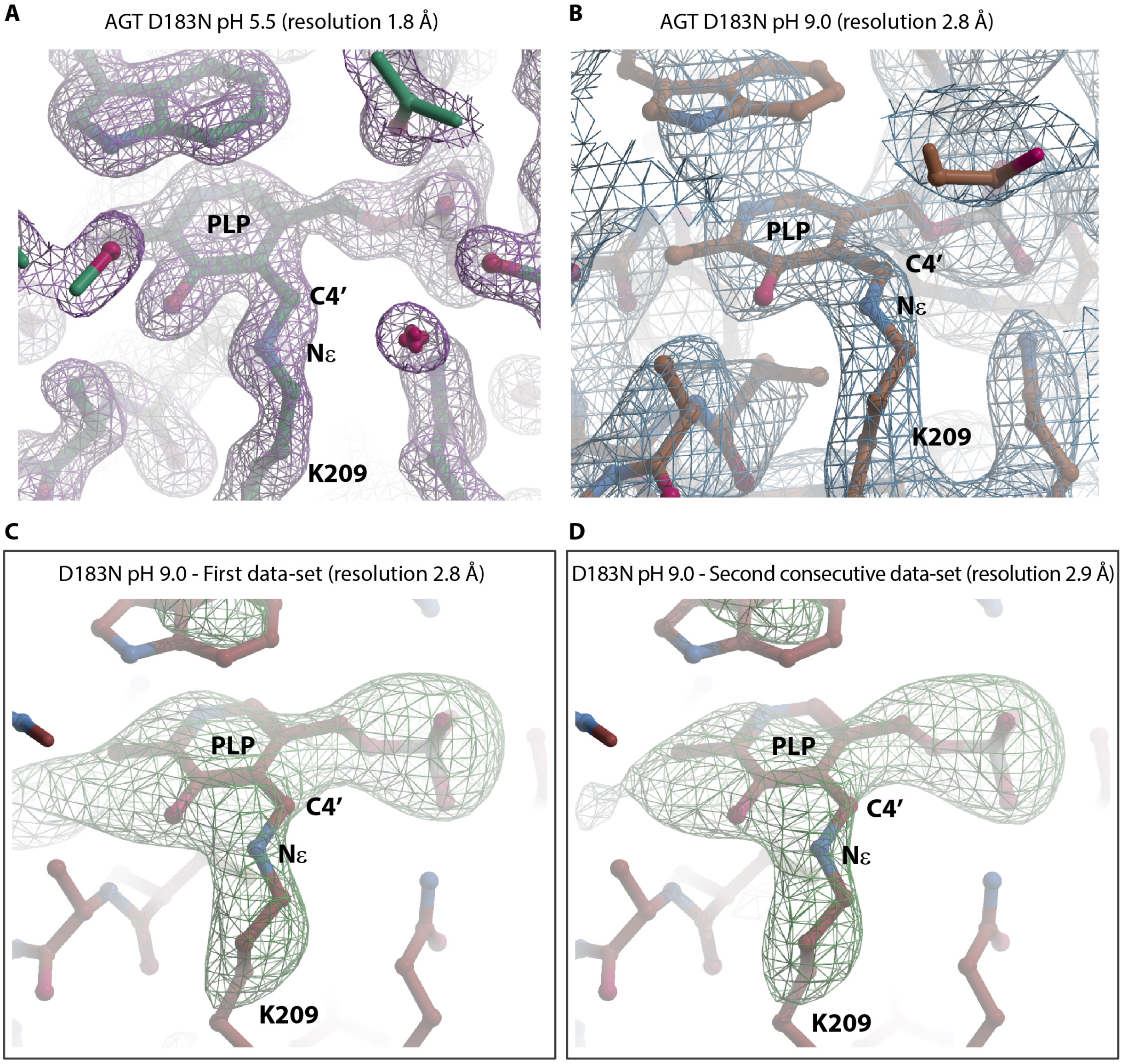

Figure S4. A) and **B)** Electron density maps ($2F_o$-F_c contoured at 1.5 σ) for the structure of the mutant AGT D183N obtained at pH 5.5 (1.8 Å resolution; *green* sticks; PDB id 5LUC) and at pH 9.0 (2.8 Å resolution; *dark red* sticks; PDB id 5OFY) respectively. In both cases a continuous density was observed in the region corresponding to the Schiff base bond between PLP-C4' and the amino group of Lys-209. **C)** and **D)** for the D183N pH 9.0 crystal, two consecutive 180 degrees datasets (2x180 frames with 1 degree of oscillation) were collected at the ELETTRA XRD1 beam line, at the same wavelength (1.0 Å) of the wild type. Then the data were analyzed as two datasets of 180 frames each, similarly to the wild type Early and Late dataset discussed in the work. No photo induced reduction of the internal aldimine was observed for the mutant, as evident by comparing the F_o-F_c maps (contoured level = 3.5 σ) calculated by omitting PLP and Lys-209 side chain, in the first and second consecutive dataset (2.8 and 2.9 resolution respectively). The second data set was not deposited.

Supplementary Video S5

Proposed reaction mechanism (from the internal to the external aldimine) between L-Ala and human AGT. The intermediate conformation between the modeled stable states (internal aldimine, geminal diamine, external aldimine) were generated using the MoMA-LigPath server and energy minimized.

Supplementary Figure S6

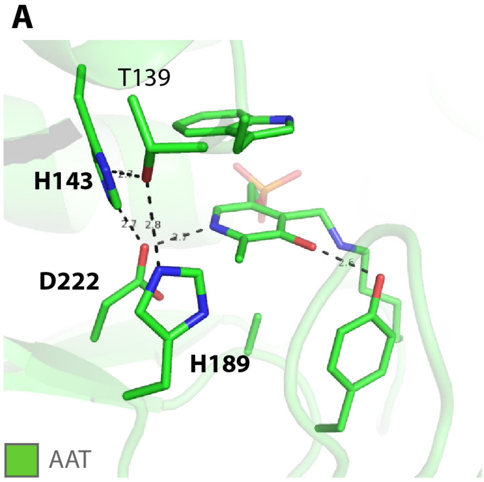

Enzyme	k_{cat} (s^{-1})	K_M (L-Asp) (μM)	k_{cat}/K_M, ($M^{-1} \cdot s^{-1}$, x10^{-3})
Wild-type	46.7 ± 3.1	384 ± 76	121
D222T	0.61 ± 0.17	110 ± 33	5.5
H143L	23.5 ± 1.9	374 ± 110	62
H143L:H189L	12.4 ± 0.9	340 ± 84	36
H143F:H189L	0.94 ± 0.1	66 ± 14	14

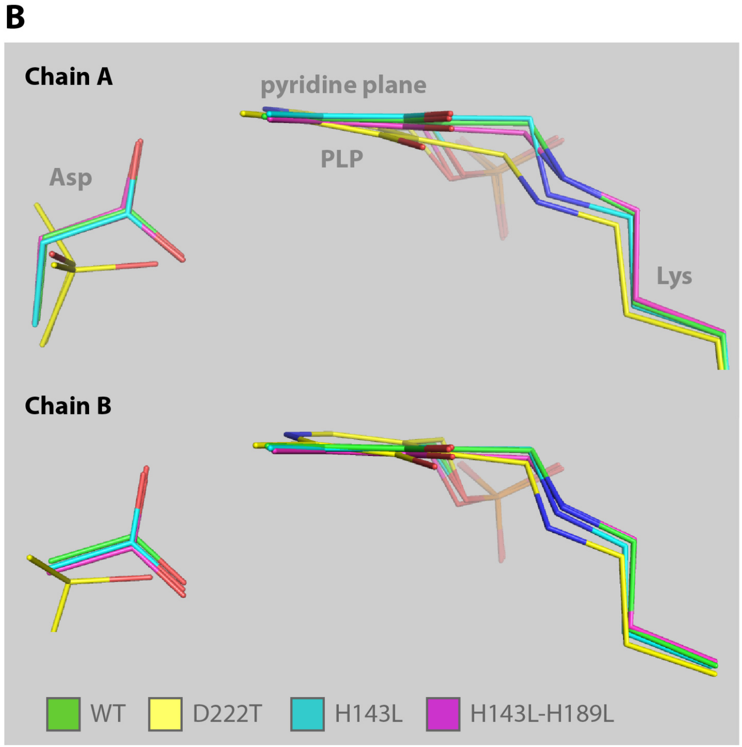

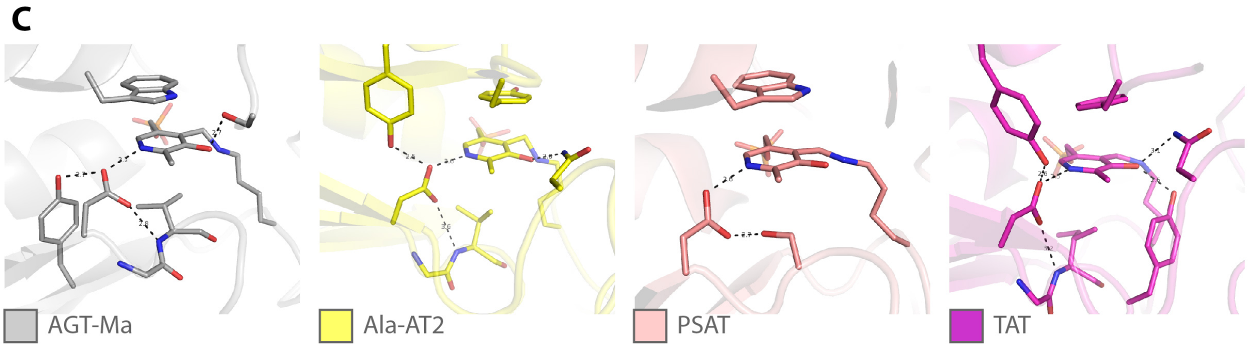

Figure S6. Second shell residues anchoring the conserved aspartate. **A)** The extended H-bond network of AAT from *Sus scrofa*. **B)** Superposition of the wt and the mutants structures (PDB id: 5TOQ, wt; 5TOR, D222T; 5TON, H143L; 5TOT, H143l-H189L) showing the change in the position of the PLP position for chain A and B [22]. The kinetic parameters as reported in the work are also shown [22]. **C)** Comparison of the second shell residues for different aminotransferase enzymes showing that these residues are not conserved. From left to right: human alanine:glyoxylate aminotransferase major allele (AGT-Ma, this work); human alanine aminotransferase 2 (PDB id: 3IHJ, unpublished); phosphoserine aminotransferase from *Bacillus alcalophilus* (PDB id: 2BHX) [9]; human tyrosine aminotransferase (PDB id: 3DYD, unpublished)
